# Androgen Receptor-Activated Enhancers Simultaneously Regulate Oncogene *TMPRSS2* and lncRNA *PRCAT38* in Prostate Cancer

**DOI:** 10.3390/cells8080864

**Published:** 2019-08-09

**Authors:** Zikai Chen, Xuhong Song, Qidong Li, Lingzhu Xie, Tangfei Guo, Ting Su, Chang Tang, Xiaolan Chang, Bin Liang, Dongyang Huang

**Affiliations:** Department of Cell Biology and Genetics, Shantou University Medical College, Shantou, Guangdong 515041, China

**Keywords:** *TMPRSS2*, *PRCAT38*, enhancer, chromatin looping

## Abstract

Prostate cancer is a common carcinoma in males, the development of which involves the androgen receptor (AR) as a key regulator. AR transactivation induces the high expression of androgen-regulated genes, including transmembrane protease serine 2 (*TMPRSS2*) and long noncoding RNA prostate cancer-associated transcript 38 (*PRCAT38*). *PRCAT38* and *TMPRSS2* are both located on chromosome 21, separated by a series of enhancers. *PRCAT38* is a prostate-specific long noncoding RNA that is highly expressed in cancer tissue as compared to normal tissue. Here, we show chromatin looping by enhancers E1 and E2 with the promoters for *PRCAT38* and *TMPRSS2*, indicating the co-regulation of *PRCAT38* and *TMPRSS2* by the same enhancers. The knockout of enhancer E1 or E2 simultaneously impaired the transcription of *PRCAT38* and *TMPRSS2* and inhibited cell growth and migration. Moreover, the loop formation and enhancer activity were mediated by AR/FOXA1 binding and the activity of acetyltransferase p300. Our findings demonstrate the utilization of shared enhancers in the joint regulation of two oncogenes in prostate cancer cells.

## 1. Introduction

Prostate cancer is the most common cancer in males worldwide. It is driven by androgen, thus, androgen deprivation therapy is effective for the first line of treatment [1]. However, nearly all patients progress to castration resistance, which is accompanied with metastasis [2,3]. The lethality of prostate cancer in men is mainly due to metastasis [4], with an average survival of about 3 years [5]. Thus, it is important to understand the regulatory mechanisms of metastasis-associated genes. The transmembrane protease serine 2 gene (*TMPRSS2*) is located on chromosome 21, 21q22.3, and encodes for a protein of 492 amino acids. It is expressed in prostatic epithelium, kidney, pancreas and colon tissues [6]. *TMPRSS2* is an androgen-regulated gene that is overexpressed in prostate cancer tissue and is specifically involved in cancer cell invasion and metastasis [7]. Additionally, the 5’ untranslated region of *TMPRSS2* tends to fuse with E26 transformation-specific (ETS) family members through chromosome rearrangement, resulting in the overexpression of ETS genes and poor prognosis in prostate cancer [8,9].

Long noncoding RNAs (lncRNAs) are RNAs longer than 200 nucleotides, without the capacity of coding proteins. LncRNAs play important regulatory roles in both normal and cancer cells, including but not limited to chromosomal silencing [10], chromatin remodeling [11], protein localization [12], and tumor suppression [13]. LncRNA *PRCAT38* is specifically overexpressed in prostate cancer, and its expression is positively correlated with *TMPRSS2* expression within tumor samples [14]. Recent studies have revealed that *PRCAT38* can promote the proliferation and migration of PC3 and DU145 prostate cancer cell lines, acting as a “sponge” to compete for miRNAs that target EZH2 [15]. However, the mechanism underlying the regulation of *PRCAT38* in prostate cancer remains unknown.

*PRCAT38* and *TMPRSS2* are both located on chromosome 21q22.3, 70 kb away from each other. Bioinformatic analysis revealed multiple regions, between the two genes, that are enriched with H3K4me1 and H3K27ac, histone marks of active enhancers [16]. Enhancers are DNA elements that regulate the gene expression independent of direction and location [17,18] and are functionally associated with cancer development [19,20]. Enhancers are sequence-specific and contain transcription factor binding sites. They can be linked to and regulate gene promoters through chromatin looping under certain conditions, thus regulating distant target genes [21,22]. Enhancer-promoter interactions are mediated by transcription cofactors, cohesion and DNA binding proteins such as CTCF or YY1 [22] and required for the appropriate regulation of target genes [23]. Besides H3K4me1 and H3K27ac, the transcription coactivator CBP/p300 is also a mark of active enhancers [24]. In addition, RNA polymerase II (Pol II) also binds at enhancer regions to generate enhancer RNA (eRNA), which is also an indicator of active enhancers [19].

We recently characterized CYR61 enhancers that play important roles in promoting cell migration during colon cancer progression [25]. In this study, to better understand enhancer regulation in cancer, we show the epigenetic co-regulation of *TMPRSS2* and *PRCAT38* via eRNA transcription, enhancer-promoter interaction, and gene regulation by transcription factors, revealing a co-regulatory function of two enhancers, denoted by E1 and E2 in the transcription of *PRCAT38* and *TMPRSS2*. In addition, the enhancer activity is mediated by AR/FOXA1 binding and the acetyltransferase p300. This study broadens the horizon of enhancer regulation on prostate cancer-related genes.

## 2. Material and Methods

### 2.1. Cell Culture

Human prostate cancer cell lines, LNCaP clone FGC (CVCL_1379) and VCaP (CVCL_2235), were purchased from the Cell Bank of the Chinese Academy of Sciences. LNCaP cells were cultured in an RPMI1640 medium (Gibco, Thermo Fisher Scientific, Shanghai, China) supplemented with 10% FBS (Gibco). VCaP cells were cultured in a DMEM medium (Gibco) supplemented with 10% FBS. Cells were cultured at 37 °C with 5% CO_2_. For androgen stimulation assays, cells were cultured in phenol red-free medium supplemented with charcoal-stripped FBS (Gibco) for 3 days and then treated with 10 nM DHT (dissolved in ethanol (Eth), MedChemExpress (MCE, Shanghai, China) for 6 h. Androgen inhibition was accomplished by the addition of 10 μM bicalutamide (dissolved in dimethylsulfoxide (DMSO), MCE) for 4 h, followed by 10 nM DHT for 6 h. p300 inhibition was performed by treating LNCaP cells with 10 μM C646 (dissolved in DMSO, MCE) for 6 h. All human cell lines have been authenticated using STR profiling within the last three years and the cells are free of mycoplasma.

### 2.2. Cell Proliferation Assay

LNCaP cells were transfected with ASO or knockout plasmids in a 6-well plate according to the manufacturer’s instructions with minor modification. Briefly, cells (1.9 mL) were inoculated one day before transfection. A total of 2 μL of 10 μM ASO was added to 92 μL Opti-MEM and mixed with 6 μL of Lipofectamine RNAiMAX reagent (Invitrogen, 13778100, Shanghai, China) while 1 μg plasmid in 94 μL Opti-MEM was mixed with 6 μL of FuGENE HD transfection reagent (Promega, E2312, Beijing, China). The mixture was incubated at room temperature for 5 min and added to the wells dropwise. At 48 h, after transfection, the images were taken under the microscope. The cells were then trypsinized and counted by hemocytometer (Qiujing Biochemical Reagent & Instruments Co., Ltd., Shanghai, China).

### 2.3. RNA Extraction, RT-PCR and Quantitative PCR

Total RNA was isolated using Trizol (Takara, No.9109, Dalian, China) according to the manufacturer’s instructions. Reverse transcription was performed using a PrimeScript™ RT reagent kit with a gDNA Eraser (Takara, RR047A). Quantitative real-time PCR was performed using SYBR Green Master Mix (Vazyme, Q131-02, Nanjing, China) on a QuantStudio 12K Flex Real-Time PCR System (Thermo Fisher Scientific, Shanghai, China). Primer sequences are shown in Supplementary Table S1. The relative expression level of the target genes was normalized to that of GAPDH.

### 2.4. Western Blot Assay

Cells were lysed in a RIPA buffer supplemented with a protease inhibitor (Millipore, 539134, Guangzhou, China). Protein concentration was quantified using a BCA kit (Beyotime Biotechnology, P0009, Shanghai, China). Protein extracts were boiled and then separated on SDS–PAGE gels followed by blotting on a polyvinylidene difluoride membrane (Millipore, IPVH00010, Guangzhou, China). Antibodies used for western blots were as follows: anti-FOXA1 (Thermo Fisher Scientific, PA5-27157, Shanghai, China), anti-β-actin (Santa Cruz, sc-130656, CA, USA), anti-H3K27ac (Thermo Fisher Scientific, 720096, Shanghai, China). Secondary antibodies were HRP-conjugated goat anti-mouse IgG (Invitrogen, 31430, Shanghai, China) and peroxidase-conjugated goat anti-rabbit IgG (Millipore AP132P, Guangzhou, China).

### 2.5. ChIP-seq and GRO-seq Data Analyses

Raw data for ChIP-seq (GEO accession ID: GSM353634, GSM353626, GSM1902615; GSM353631, GSM353620, GSM2827606) and GRO-seq (GEO accession ID: GSE83860, GSE84432) were downloaded from the GEO database [26,27,28]. Quality control for the reads of the fastq files was performed by FastQC. Raw data were aligned using Bowtie2 version 2.2.9 by default setting to build version HG19 of the human genome. The peak finding algorithm of the MACS version 1.4.1 was used to identify regions of ChIP-Seq and GRO-Seq enrichment. A *P*-value threshold of enrichment of 10^−5^ was used for all datasets. The density of reads was normalized by calculating RPM (reads per million mapped reads). The R package “Sushi” was used to plot the figures.

### 2.6. RNA Interference (RNAi) Assay

siRNAs were designed and purchased from GenePharma (Shanghai, China) and antisense oligonucleotides (ASO) were purchased from Takara (Dalian, China). Cells were transfected with siRNA or ASO using a Lipofectamine RNAiMAX reagent (Invitrogen, 13778100, Shanghai, China) according to the manufacturer’s protocols. Briefly, cells (1.9 mL) were inoculated one day before transfection. A total of 2 μL of 10 μM ASO or siRNA was added to 92 μL Opti-MEM and mixed with 6 μL of Lipofectamine RNAiMAX Reagent (Invitrogen, 13778100). The mixture was incubated at room temperature for 5 min and then added to the wells dropwise. At 48 h after transfection, the cells were harvested for further analysis. The sequences of siRNA and ASO are listed in Supplementary Table S3.

### 2.7. Chromatin Immunoprecipitation (ChIP)

ChIP assays were performed using a Magna ChIP™ G Chromatin Immunoprecipitation Kit (Millipore, 17-611, Guangzhou, China) as previously described [29]. Briefly, cells were fixed with 4% formaldehyde for 10 min and then scraped after washing with PBS twice. The cells were treated with a cell lysis buffer and sonicated on ice to generate 500–1000 bp DNA fragments. Then, 2–3 μg ChIP-grade antibodies against anti-Pol II (Millipore, 05-623), anti-H3K27ac (Thermo Fisher Scientific, 720096), anti-H3K4me1 (Abcam, ab8895), or anti-FOXA1 (Thermo Fisher Scientific, PA5-27157) were used to perform ChIP assays with protein A/G magnetic beads (MCE). Immunoprecipitated DNA was purified and applied to qPCR and normalized to the input DNA. The sequences of the primers are listed in Supplementary Table S2.

### 2.8. Dual-luciferase Reporter Assay

Enhancer regions were inserted into the pGL3-Promoter vector (Promega, E1751, Beijing, China). Primers used for molecular cloning are listed in Supplementary Table S4. The control pRL-SV40 Vector was purchased from Promega (E2231). All engineered constructs were verified by sequencing. Plasmid transfection was performed with a FuGENE HD transfection reagent (Promega, E2312) according to the manufacturer’s protocol. Luciferase activity was determined by the dual-luciferase reporter assay system (Promega, E1910) 48 h after transfection.

### 2.9. Knockout of Enhancer Regions

sgRNA targeting enhancer regions were designed using a webtool: http://crispor.tefor.net/ [30]. Screening in a cell-free system was performed using GenCrispr sgRNA Synthesis Kit (GenScript L00694, Nanjing, China) and GenCrispr sgRNA Screening Kit (GenScript L00689, Nanjing, China) to determine the efficacy of sgRNAs. LNCaP cells were transfected with lentiCas9-Blasticidin lentivirus (Genomeditech, Shanghai, China) prior to transfection with plasmids LentiGuide-EF1-ZsGreen1-T2A-Puro or pGMLV-hU6-gRNA-EF1a-mCherry-WPRE (Genomeditech, Shanghai, China), containing sgRNAs targeting enhancer E1 and E2, respectively. sgRNA sequences and primer sequences for verification of enhancer knockout are listed in Supplementary Table S7. To get the clone of homozygous deletion of enhancer E2, transfected cells were diluted to 1 cell/100 μL and inoculated into 96-well plates. Single cells were visually determined under a microscope. Genomic DNA was isolated when single cells proliferated to enough cells and applied to PCR verification. PCR products were verified by Sanger Sequencing.

### 2.10. Chromosome Conformation Capture (3C) Assay

The 3C assay was performed as described previously [31,32]. Plasmids used in 3C assays were BAC clones CTD-3251N23 and RP11-671L10 (Invitrogen, Shanghai, China). Restriction enzyme HindIII was used for BAC DNA and genomic DNA digestion. Primers used are shown in Supplementary Table S5.

### 2.11. Cell Migration Assay

A cell migration assay was performed using a transwell chamber (24-well, 8 μm pore size; Corning, Guangzhou, China). Cells were treated with 10 nM ASO or knockout plasmids in a 6-well plate for 48 h, and 10^5^ cells were transplanted into the chambers. After 48 h, the migrated cells were stained with crystal violet and counted in 5 random fields.

### 2.12. Statistical Analysis

A paired-samples t-test was used to determine if the difference between the two groups of data was significant (*P* < 0.05 was considered significant). All data shown were determined with at least three independent experiments and presented as the mean ± standard deviation.

## 3. Results

### 3.1. PRCAT38 is an Androgen-Regulated lncRNA that Modulates Cell Growth and Migration in Prostate Cancer

**Figure 1:**
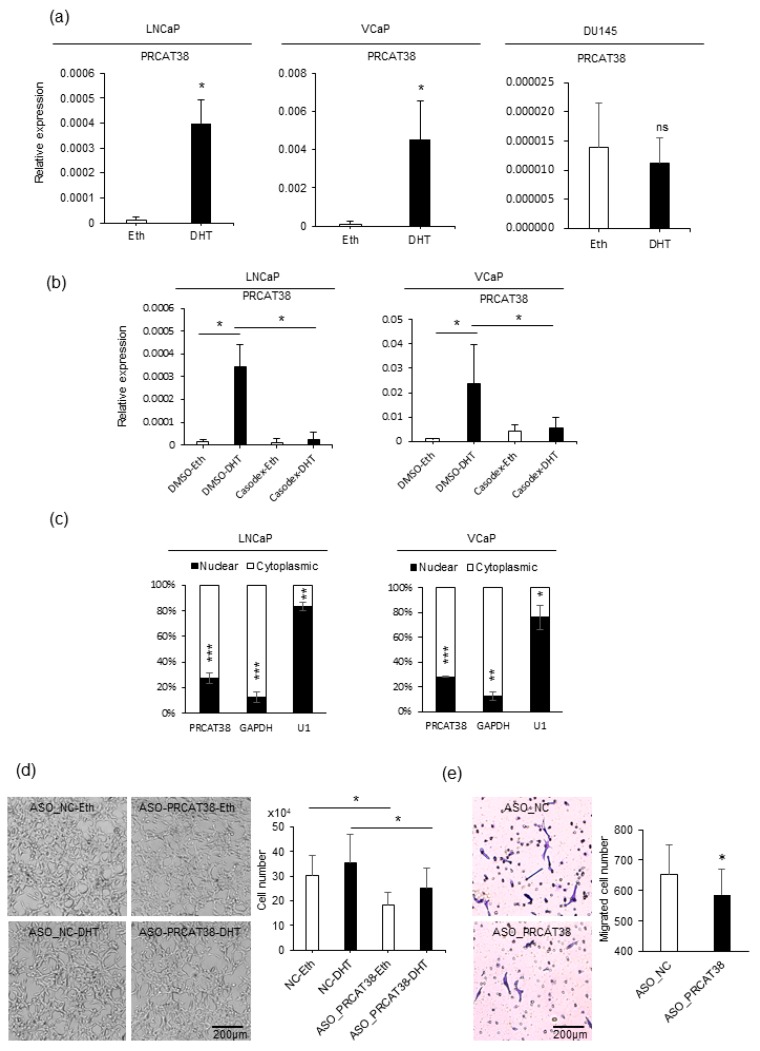
*PRCAT38* is an androgen-regulated, prostate cancer-specific lncRNA that modulates cell growth and cell migration. (**a**) *PRCAT38* is highly induced by DHT in LNCaP and VCaP cells, but not DU145 cells. (**b**) Androgen induction of *PRCAT38* is inhibited by the androgen receptor antagonist bicalutamide in LNCaP and VCaP cells. (**c**) Nuclear-cytoplasmic fractionation showing the subcellular location of *PRCAT38*, *GAPDH* and *U1* in LNCaP and VCaP cells. (**d**) growth of LNCaP cells before and after *PRCAT38* knockdown. (**e**) Migration of LNCaP cells before and after *PRCAT38* knockdown. Data are shown as the mean ± SD (n = 3). *: *P* < 0.05, **: *P* < 0.01, ***: *P* < 0.001, ns: not significant.

To explore strongly the oncogenic lncRNAs and their lineage association in prostate cancer, we searched for uncharacterized transcripts that were highly expressed in prostate cancer samples in the MiTranscriptome project [33]. In more than 20 types of tissues, *PRCAT38* was solely expressed in both normal and tumor prostate samples (Supplementary Figure S1A). Importantly, it was significantly up-regulated in cancer when compared to normal prostate tissue (Supplementary Figure S1B). LncRNAs that play crucial roles in prostate carcinogenesis tend to be AR-dependent and androgen-upregulated [34]. Thus, we investigated the expression of *PRCAT38* before and after dihydrotestosterone (DHT) treatment in LNCaP, VCaP and DU145 cells. The results showed a significant increase in *PRCAT38* expression after DHT induction in LNCaP and VCaP cells, but no change in DU145 cells (Figure 1a). To further validate that the expression of *PRCAT38* was AR-dependent, we pretreated the cells with the androgen receptor antagonist bicalutamide (Casodex) before adding DHT. DHT-induced transcription of *PRCAT38* was inhibited by bicalutamide, as was *TMPRSS2* (Figure 1b, Supplementary Figure S1C). Next, we inspected the sub-cellular location and function of *PRCAT38*. Nuclear-cytoplasmic fractionation followed by qPCR showed that *PRCAT38* is mainly located within the cytoplasm (Figure 1c), and cell proliferation and migration of LNCaP cells were decreased upon silencing of *PRCAT38* (Figure 1d, e). This was consistent with a previous study examining *PRCAT38* overexpression in DU145 cells [15]. In short, these results indicate that *PRCAT38* is an AR-regulated, prostate lineage-specific transcript that is over-expressed in cancer.

### 3.2. AR binds to Enhancers between TMPRSS2 and PRCAT38, to Recruit RNA Pol II and H3K27ac to Initiate Transcription

A recent study on clinical samples showed that the expression of *PRCAT38* is highly correlated with that of *TMPRSS2* [14]. *PRCAT38* is located 70 kb upstream of *TMPRSS2*. The two genes are separated by a genomic region highly enriched in H3K4me1 and H3K27ac, but with a low content of H3K4me3 (Supplementary Figure S2A), indicating the existence of a series of enhancers in between the *TMPRSS2* and *PRCAT38* gene loci. In order to characterize the enhancer regions, we integrated the H3K4me1 and H3K27ac ChIP peaks with GRO-seq peaks in LNCaP and VCaP cells [26,27,28], resulting in the identification of 6 potential enhancers (Supplementary Figure S2A).

**Figure 2:**
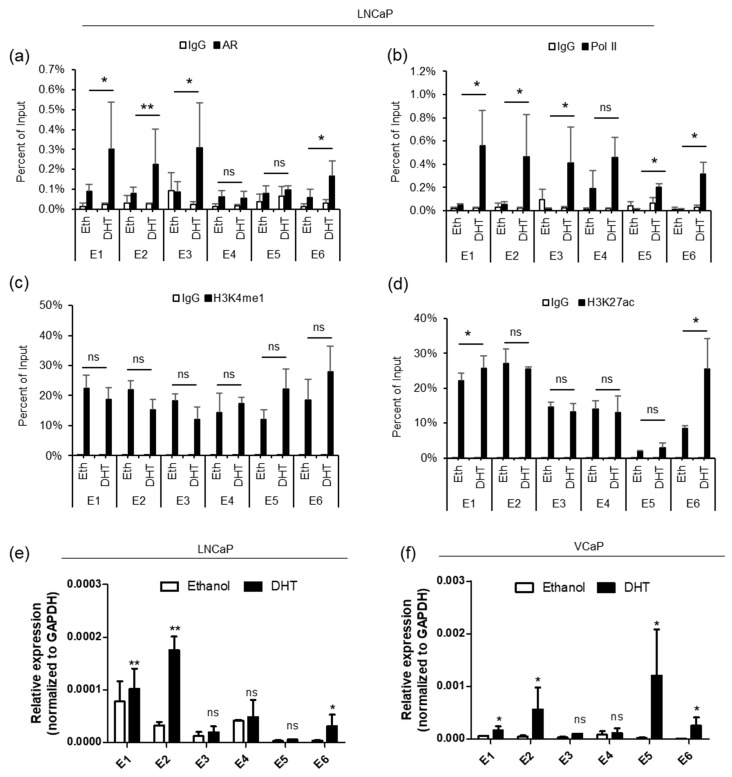
AR binds to the enhancers, between *TMPRSS2* and *PRCAT38*, recruiting Pol II and H3K27ac to activate the enhancers. (**a**–**d**) ChIP-qPCR in LNCaP cells before and after DHT treatment showing AR, RNA polymerase II, H3K4me1 and H3K27ac enrichment over the potential enhancer regions. Data are shown as the mean ± SD (n = 3). * indicates a significant change of the DHT treatment over the vehicle. (**e**–**f**) eRNA transcription from potential enhancers before and after DHT treatment in LNCaP and VCaP. *: *P* < 0.05, **: *P* < 0.01, ns: not significant.

Next, we performed ChIP-qPCR in LNCaP cells to determine the enrichment of AR, Pol II and histone modification marks on individual enhancers and the *PRCAT38* promoter before and after DHT treatment. The results showed that before DHT induction, there was almost no AR or Pol II binding at the enhancers (Figure 2a,b), although H3K4me1 and H3K27ac were significantly enriched at the enhancer regions (Figure 2c,d). After DHT treatment, AR binding was increased at enhancers E1, E2, E3 and E6, but not at E4 and E5 (Figure 2a). Pol II enrichment was increased across all regions except for E4 (Figure 2b). There was not much change for H3K4me1 enrichment before and after DHT treatment across all regions (Figure 2c). On the other hand, H3K27ac enrichment only increased at enhancers E1 and E6 (Figure 2d). We observed similar results in VCaP cells (Supplementary Figure S2B–E). AR enrichment was not affected at the promoter of *PRCAT38*, but interestingly, H3K27ac increased and PolII was recruited to the *PRCAT38* promoter region (200 bp upstream of *PRCAT38*) after DHT treatment (Supplementary Figure S3A–D).

In addition to H3K4me1 and H3K27ac, eRNA transcription is also an indicator of active enhancers [19]. We next examined eRNA transcription from the 6 potential enhancer regions, in LNCaP and VCaP cells, following DHT treatment. The transcriptional activity from enhancers E1, E2 and E6 was highly inducible by DHT. On the contrary, transcription from E3-E5 occurred at low levels and was not activated by androgen (Figure 2e,f). These results are consistent with the ChIP results, revealing poised but androgen-dependent enhancers near the promoters of the two genes. The transcriptional activity of enhancers in VCaP cells was similar to that of LNCaP cells, except that the transcription of eRNA from E5 was inducible in VCaP, but not in LNCaP cells (Figure 2e,f), in agreement with the GRO-seq data (Supplementary Figure S2A). These results suggest that enhancers E1, E2 and E6 are in a poised state without androgen treatment, but can be activated when Pol II and H3K27ac are recruited upon DHT induction.

### 3.3. Enhancer E1 is Responsive to Androgen Induction and Regulates both TMPRSS2 and PRCAT38 via Interacting with E2 through Chromatin Looping

**Figure 3:**
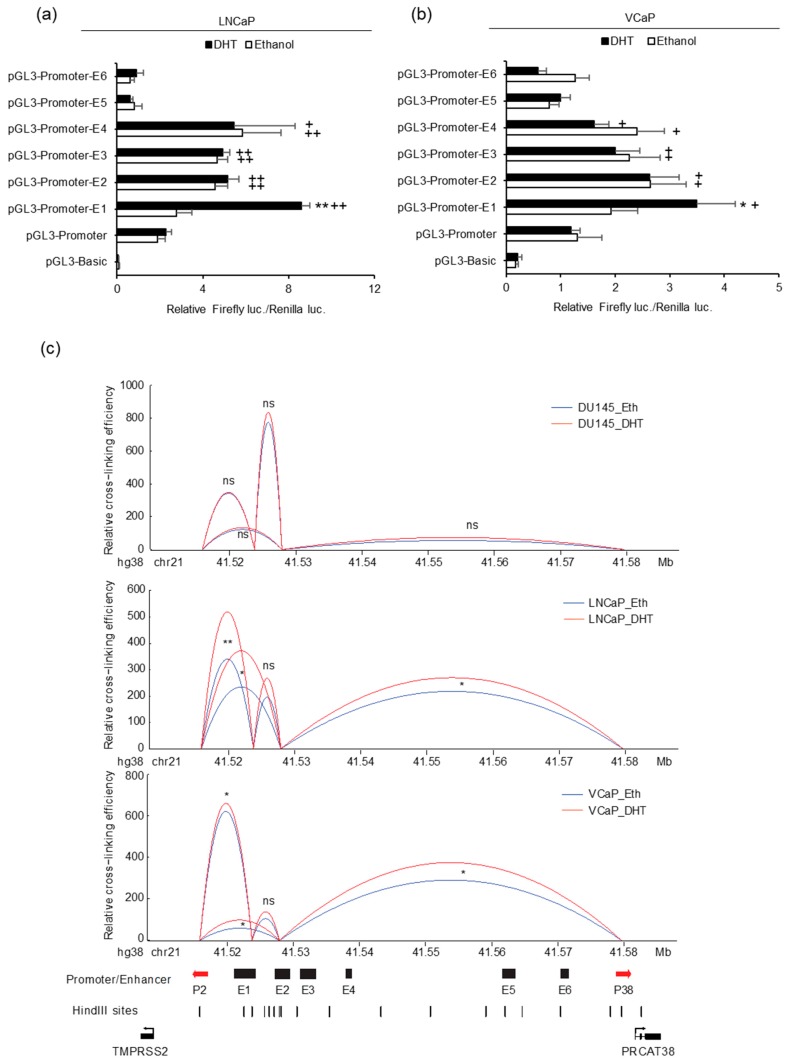
Enhancers E1 and E2 mediate *TMPRSS2* and *PRCAT38* gene regulation through chromatin looping. (**a**–**b**) Dual-luciferase reporter assays to determine enhancer activity in LNCaP and VCaP cells. Data are shown as mean ± SD (n = 3). * indicates a significant difference between the vehicle and DHT treatment, + indicates a significant difference between each construct and the pGL3-Promoter. (**c**) 3C-qPCR analysis revealing chromatin looping between enhancer regions and promoters of *TMPRSS2* and *PRCAT38* in DU145, LNCaP and VCaP cells (peak values of the loops are the mean of 3 biological replicates). */+: *P* < 0.05, **/++: *P* < 0.01, ns: not significant.

In order to further characterize the activity of the 6 potential enhancers, we individually cloned each enhancer into the luciferase reporter vector pGL3-Promoter and detected luciferase activity. We expected strong activity from the enhancers near the promoters of the two genes. However, the results showed that only the ones close to the *TMPRSS2* promoter increased the promoter activity and that only E1 was responsive to DHT (Figure 3a,b). This result indicated that E6 may not directly function to enhance the transcription of *PRCAT38*. Considering the DHT-induced *PRCAT38* transcription and the activation of only enhancer E1 after DHT treatment, we predicted that there would be chromatin looping bringing *PRCAT38* in proximity to enhancer E1. To this end, we employed 3C to detect the chromatin looping among the enhancers and promoters of *TMPRSS2* and *PRCAT38*. Enhancer E1 interacted with the *TMPRSS2* promoter as expected, as it was previously found to be crucial for the regulation of *TMPRSS2* [35]. The interaction frequency between enhancer E1 and the *PRCAT38* promoter was low; however, there was a frequent interaction between enhancer E2 and the *PRCAT38* promoter. More importantly, the interaction ratio was further increased by DHT treatment. Chromatin also looped between enhancer E1 and E2, which served as a bridge for enhancer E1 to regulate *PRCAT38*. The interaction between enhancers and promoters in DU145 was much lower and not affected by DHT treatment (Figure 3c). This result indicates that E1 and E2 mediate the regulation of *TMPRSS2* and *PRCAT38* expression through chromatin looping directly and indirectly, respectively.

**Figure 4:**
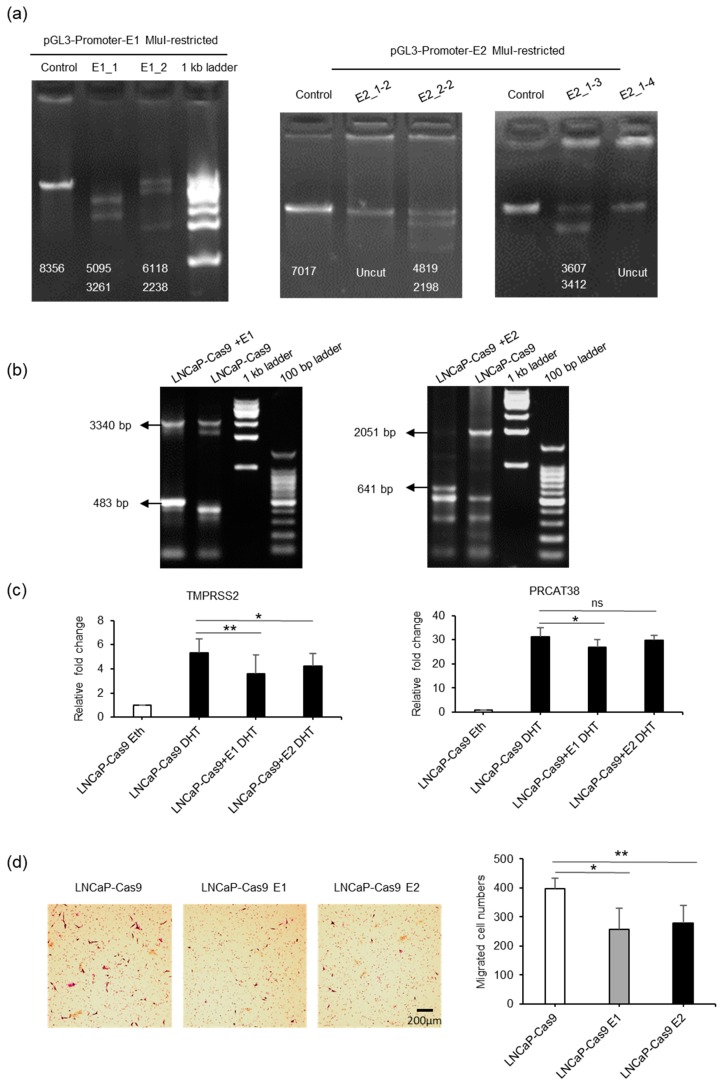
Knockout of enhancer E1 or E2 downregulates the transcription of *TMPSS2* and *PRCAT38*. (**a**) Determination of the efficacy of sgRNAs using MluI-restricted plasmids as templates in a cell-free system (Figure S4A). Numbers on the gel pictures indicate the expected size of the products after Cas9-mediated DNA digestion. (**b**) Determination of efficacy of sgRNAs after transfection of dual-sgRNA plasmids into LNCaP-Cas9 cells. The numbers on the gel pictures indicate PCR products before and after knockout. (**c**) Knockout of enhancer E1 or E2 in a subpopulation of cells downregulates *TMPRSS2* and *PRCAT38* transcription after DHT treatment. (**d**) Knockout of enhancer E1 or E2 impairs the migration of LNCaP cells. Data are shown as the mean ± SD (n = 3), *: *P* < 0.05, **: *P* < 0.01, ns: not significant.

Next, we performed knockout experiments for enhancers E1 and E2 in LNCaP cells (Figure 4a–b, Supplementary Figure S4A,B). When the enhancer regions were knocked out, the transcription of *TMPRSS2* and *PRCAT38* was significantly downregulated (Figure 4c, Supplementary Figure S4C). Moreover, knockout cells were much less invasive (Figure 4d) and grew more slowly than the control cells (Supplementary Figure S4D). These results further support the hypothesis of the co-regulation of two oncogenes by the same enhancers.

### 3.4. FOXA1 is Recruited by the AR to the Enhancers and Regulates the Expression of TMPRSS2 and PRCAT38

**Figure 5:**
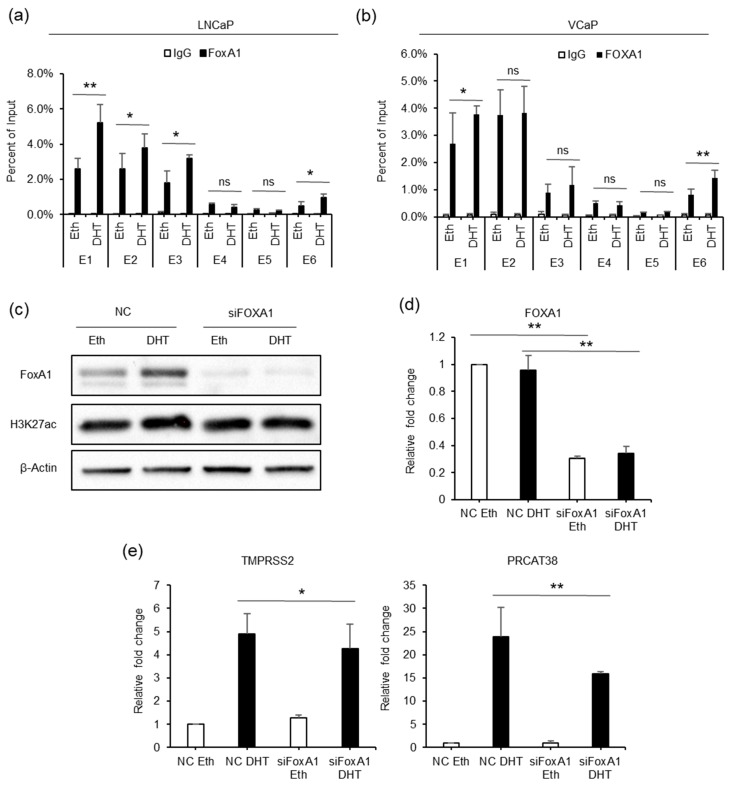
FOXA1 is recruited by the AR to the enhancers and regulates the transcription of *TMPRSS2* and *PRCAT38*. (**a**,**b**) ChIP-qPCR before and after DHT treatment showing FOXA1 enrichment over the enhancer regions in LNCaP and VCaP. (**c**) Western blot showing the protein level of FOXA1 and H3K27ac after FOXA1 knockdown with or without DHT treatment. (**d**–**e**) qPCR showing the down-regulation of *FOXA1*, *TMPRSS2* and *PRCAT38* after FOXA1 knockdown with or without DHT treatment. Data are shown as mean ± SD (n = 3).

To better understand the epigenetic regulation of *TMPRSS2* and *PRCAT38*, we used the transcription factor affinity prediction (TRAP) web tools to search for transcription factors whose binding motifs are overrepresented in the 6 enhancers [36]. The forkhead box protein A1 (FOXA1) motif was significantly enriched and ranked first (Supplementary Table S6). The results showed that FOXA1 binding was detected at enhancers E1, E2, E3 and E6, but not at enhancers E4 and E5, similar to the AR binding pattern. FOXA1 enrichment was significantly increased after DHT treatment in both LNCaP and VCaP cells (Figure 5a,b). Moreover, FOXA1 knockdown resulted in the transcriptional inhibition of *TMPRSS2* and *PRCAT38* in LNCaP (Figure 5d,e) and VCaP (Supplementary Figure S5A) cells. The FOXA1 protein level was increased after DHT treatment (Figure 5c), though FOXA1 mRNA was not affected (Figure 5d). The inconsistency of the protein and mRNA levels may be due to post-transcriptional regulation. On the other hand, FOXA1 overexpression did not promote the transcription of *TMPRSS2* or *PRCAT38* (Supplementary Figure S5B). This suggested that it was FOXA1 binding, not the total amount of FOXA1, that regulates the transcription of the two genes. These results imply that FOXA1 recruited by AR to the enhancers plays a regulatory role in the transcription of *TMPRSS2* and *PRCAT38*.

### 3.5. FOXA1 is Required for Enhancer Activity and Chromatin Looping between Enhancers and Promoters of TMPRSS2 and PRCAT38

**Figure 6:**
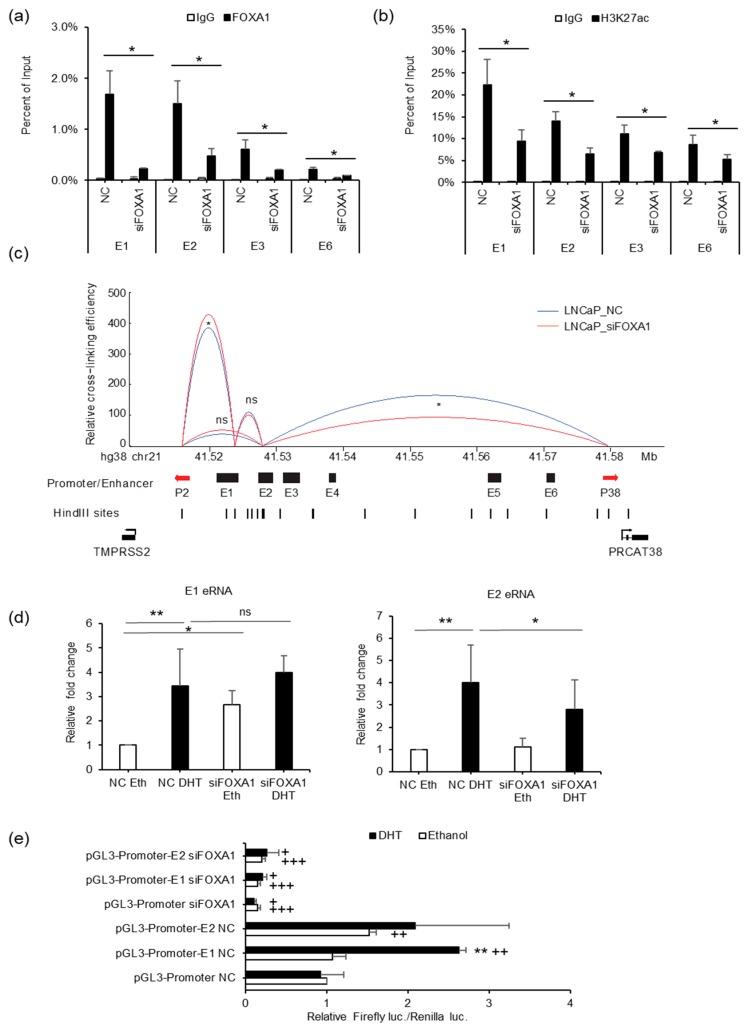
FOXA1 is required for enhancer activity and chromatin looping between enhancers and promoters of *TMPRSS2* and *PRCAT38*. (**a**,**b**) ChIP-qPCR in LNCaP cells before and after FOXA1 knockdown showing FOXA1 and H3K27ac enrichment over the enhancer regions. (**c**) 3C-qPCR analysis of the looping between enhancers and promoters before and after FOXA1 knockdown in LNCaP (peak values of the loops are the mean of 3 biological replicates). (**d**) eRNA transcription from enhancers E1 and E2 before and after FOXA1 knockdown in LNCaP. (**e**) Dual-luciferase reporter assay to determine enhancer activity of E1 and E2 before and after FOXA1 knockdown in LNCaP. Data are shown as the mean ± SD (n = 3); * indicates a significant difference between vehicle and DHT treatment, + indicates significant difference between respective construct and pGL3-promoter. */+: *P* < 0.05, **/++: *P* < 0.01, ns: not significant.

To elucidate the function of FOXA1 in the regulation of *TMPRSS2* and *PRCAT38*, we performed ChIP-PCR for FOXA1, H3K27ac, AR, and H3K4me1 after FOXA1 knockdown. FOXA1 binding was sharply decreased at enhancers E1, E2, E3 and E6 upon silencing (Figure 6a). H3K27ac enrichment was also reduced after FOXA1 knockdown (Figure 6b), indicating the impairment of enhancer activity with a loss of FOXA1. Such an impairment may be ubiquitous as the induction of H3K27ac by DHT was repressed by FOXA1 knockdown (Figure 5c). On the contrary, H3K4me1 enrichment was not affected by the FOXA1 loss (Supplementary Figure S5C). In addition, AR binding was not affected by the FOXA1 knockdown (Supplementary Figure S5D), further supporting that FOXA1 was recruited by AR to the enhancers, but not the other way around, and the chromatin loop between enhancer E2 and the *PRCAT38* promoter, as well as the one between enhancer E1 and the *TMPRSS2* promoter, were both impaired after the removal of FOXA1 (Figure 6c). We also looked into the transcription of eRNAs from enhancer E1 and E2 and found that only E2 eRNA was transcriptionally inhibited by FOXA1 knockdown under DHT treatment conditions (Figure 6d). Dual luciferase assays also showed the function of FOXA1 in the activation of enhancer E1 and E2 (Figure 6e).

### 3.6. p300 Inhibition Impedes Enhancer-FOXA1 Binding and Chromatin Looping

**Figure 7:**
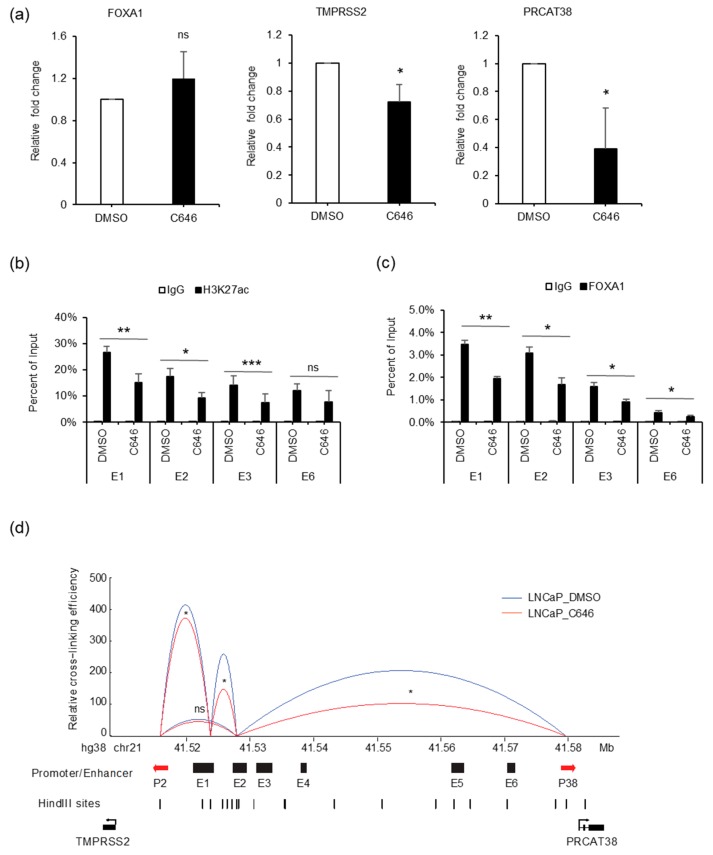
p300 inhibition impedes FOXA1 binding at the enhancers and chromatin looping between enhancers E1, E2 and the two promoters. (**a**) qPCR showing the change in expression of *FOXA1*, *TMPRSS2* and *PRCAT38* before and after C646 treatment in LNCaP. (**b**–**c**) ChIP-qPCR in LNCaP cells before and after C646 treatment showing H3K27ac and FOXA1 enrichment over the enhancer regions. Data are shown as the mean ± SD (n = 3). (**d**) 3C-qPCR analysis of the looping between enhancers and promoters before and after C646 treatment in LNCaP (peak values of the loops are the mean of 3 biological replicates). *: *P* < 0.05, **: *P* < 0.01, ns: not significant.

AR binding not only recruited FOXA1, but also increased H3K27ac enrichment at the enhancer regions. To explore the effect of H3K27ac on gene regulation, we treated cells with C646, an inhibitor of histone acetyltransferase p300. FOXA1 expression was not affected by C646 (Figure 7a, Supplementary Figure S6A). On the other hand, the transcription of *TMPRSS2* and *PRCAT38* were both down-regulated with C646 treatment (Figure 7a, Supplementary Figure S6B). H3K27ac levels at enhancers E1, E2, E3 and E6 were significantly reduced (Figure 7b), though the total amount of H3K27ac within the cells was not affected (Supplementary Figure S6A), possibly due to the redundant function of other histone acetyltransferases. Interestingly, FOXA1 binding at the enhancers was impeded after the inhibition of p300 (Figure 7c). This result indicates that FOXA1 binding was facilitated by the enrichment of H3K27ac, which was previously shown to be regulated by FOXA1 (Figure 6b), indicating a positive feedback loop for FOXA1 binding and the “writing” of H3K27ac marks. In addition, the interaction frequency between enhancers and promoters of *TMPRSS2* and *PRCAT38* was also decreased after C646 treatment (Figure 7d). On the other hand, AR binding and H3K4me1 enrichment were not affected by C646 treatment (Supplementary Figure S6C,D). eRNAs transcribed from enhancer E1 and E2 were not affected by p300 inhibition (Supplementary Figure S6E).

## 4. Discussion

Prostate cancer-associated lncRNAs have been found to function as oncogenes [34,37], participating in various signaling pathways. *PRCAT38* was found to promote cell migration and proliferation by upregulating methyltransferase EZH2 via the adsorption of miR-143-3p and miR-24-2-5p [15]. In this study, we found that *PRCAT38* is highly induced by androgen in LNCaP and VCaP cells and confirmed its ability to enhance proliferation and migration.

Four enhancers close to the *TMPRSS2* promoter can increase promoter activity, but only enhancer E1 is responsive to DHT treatment, which implies the gene regulation of *PRCAT38* by enhancer-promoter long-range chromatin interaction. Chromatin interaction via looping has been realized to be the basis of enhancer-mediated gene regulation [38,39]. The regulatory units as promoters and enhancers control their targets in a time- and space-dependent format, which is affected by the combined action of external stimuli and internal environment, exemplified by the activation of the hormonal transcription factor AR. We show that chromatin forms a loop between enhancer E1 and the *TMPRSS2* promoter, and also forms loops between enhancer E2 and promoters for both *TMPRSS2* and *PRCAT38*, as well as enhancer E1 and E2. In addition, the interaction frequencies of enhancers E1 and E2 with the promoters are regulated by the AR, FOXA1 binding and the activity of p300, indicating a set of regulatory units that simultaneously control two genes. Importantly, the results revealed that the chromatin looping existed before androgen stimulation and after FOXA1 knockdown or p300 inhibition, consistent with a comparatively stable chromatin landscape [39]. These results show direct regulation of *TMPRSS2* by enhancer E1, and indirect regulation of *PRCAT38* through E1–E2 looping, with enhancer E2 serving as a “bridge” between enhancer E1 and the *PRCAT38* promoter (Supplementary Figure S7). The knockout of enhancer E1 or E2 resulted in the downregulation of *TMPRSS2* and *PRCAT38*, further supporting a co-regulatory mechanism, which may partially explain the co-expression of the two genes in prostate tissues [14]. Only E1 and E2 out of the 6 identified enhancers are involved in the gene regulation. The reasons for cells not utilizing proximal enhancers to regulate *PRCAT38* seems perplexing, but to simultaneously control two genes using one set of regulatory units may be more efficient for the response to androgens. Functional assays for pure E1 knockout cells would better confirm the roles of enhancers on both genes, but we have thus far been unable to separate them. This may be due to low cell viability as a pure knockout colony.

AR binding to the chromatin requires transcription factors and histone modifications that open up the chromatin; on the other hand, AR binding could recruit more cofactors and transcriptional complexes [40]. We found that AR mainly binds at the enhancer regions rather than the promoter, recruiting in FOXA1, p300 and Pol II, thus activating enhancers. Interestingly, although AR does not bind to the *PRCAT38* promoter nor displays enhanced recruitment following DHT addition, Pol II and H3K27ac enrichment are both significantly increased at the promoter regions after DHT treatment. Such an increase may be fulfilled by the enhancer-promoter interaction through looping. A previous study has shown that H3K4 methylation was not required for transcriptional regulation or target gene expression [41], which is consistent with our study (Figure 2c, Supplementary Figures S2D, S5C, S6D). The enrichment at enhancer E2 and E3 is even reduced after DHT in VCaP cells. Instead, H3K27ac may be more reliable in the prediction of enhancer activity. eRNA transcription occurs at enhancer regions [42,43,44] and is a hallmark of active enhancers. eRNA transcription is associated with the expression of genes nearby, indicating a role for eRNA in gene regulation [45,46]. In our study, eRNA transcription from enhancer E2 is regulated by AR and FOXA1. Whether it functions in the regulation of *TMPRSS2* and *PRCAT38* requires further study.

Enhancer regulation of *TMPRSS2* has been extensively studied [35] and the functions of *PRCAT38* have been recently elucidated [14,15], but the link between these two genes has not been shown. Our study is the first to show the cooperation of two enhancers, E1 and E2, in regulating *TMPRSS2* and *PRCAT38*, which enables us to understand the complicated regulatory circuits of gene transcription and identifies shared enhancers of common oncogenes that can be targeted for more substantial effects in clinical trials.

## Supplementary Materials

The following are available online at https://www.mdpi.com/2073-4409/8/8/864/s1, Figure S1: PRCAT38 is androgen responsive and specifically expressed in prostate cancer samples, Figure S2: AR binds to enhancers between TMPRSS2 and PRCAT38, recruiting Pol II and H3K27ac to activate enhancers in VCaP cells, Figure S3: AR, Pol II, H3K4me1 and H3K27ac enrichment at the PRCAT38 promoter, Figure S4: Knockout of enhancer E1 or E2 downregulates transcription of TMPRSS2 and PRCAT38, Figure S5: Knockdown of FOXA1 inhibits PRCAT38 transcription, Figure S6: p300 inhibition does not affect the total amount of H3K27ac or eRNA transcription from enhancer E1 or E2, Figure S7: Schematic for simultaneous regulation of TMPRSS2 and PRCAT38 by enhancer E1 and E2, Table S1: Primer sequences for quantitative PCR, Table S2: Primer sequences for ChIP PCR, Table S3: The sequences of siRNA and ASO, Table S4: Primer sequences for molecular cloning, Table S5: Primer sequences for 3C assays, Table S6: Transcription factors predicted by TRAP web tools, Table S7: sgRNA sequences and primer sequences for verification of enhancer knockout.

## Abbreviations

AR	androgen receptor
Eth	ethanol
DHT	dihydrotestosterone
DMSO	dimethylsulfoxide
*TMPRSS2*	transmembrane protease serine 2
ETS	E26 transformation-specific
*PRCAT38*	prostate cancer associated transcript 38
FOXA1	forkhead box protein A1
Pol II	RNA polymerase II
SD	standard deviation
