# Supplementary material for: Androgen Receptor-Activated Enhancers Simultaneously Regulate Oncogene TMPRSS2 and lncRNA PRCAT38 in Prostate Cancer
Source: Cells. 2019 Aug 9;8(8):864. doi: 10.3390/cells8080864 (PMC6721761; doi:10.3390/cells8080864)
Supplement: Supplementary file 1 [file cells-08-00864-s001.zip › cells-534835-supplementary/cells-534835-Supplementary Figures.pdf]

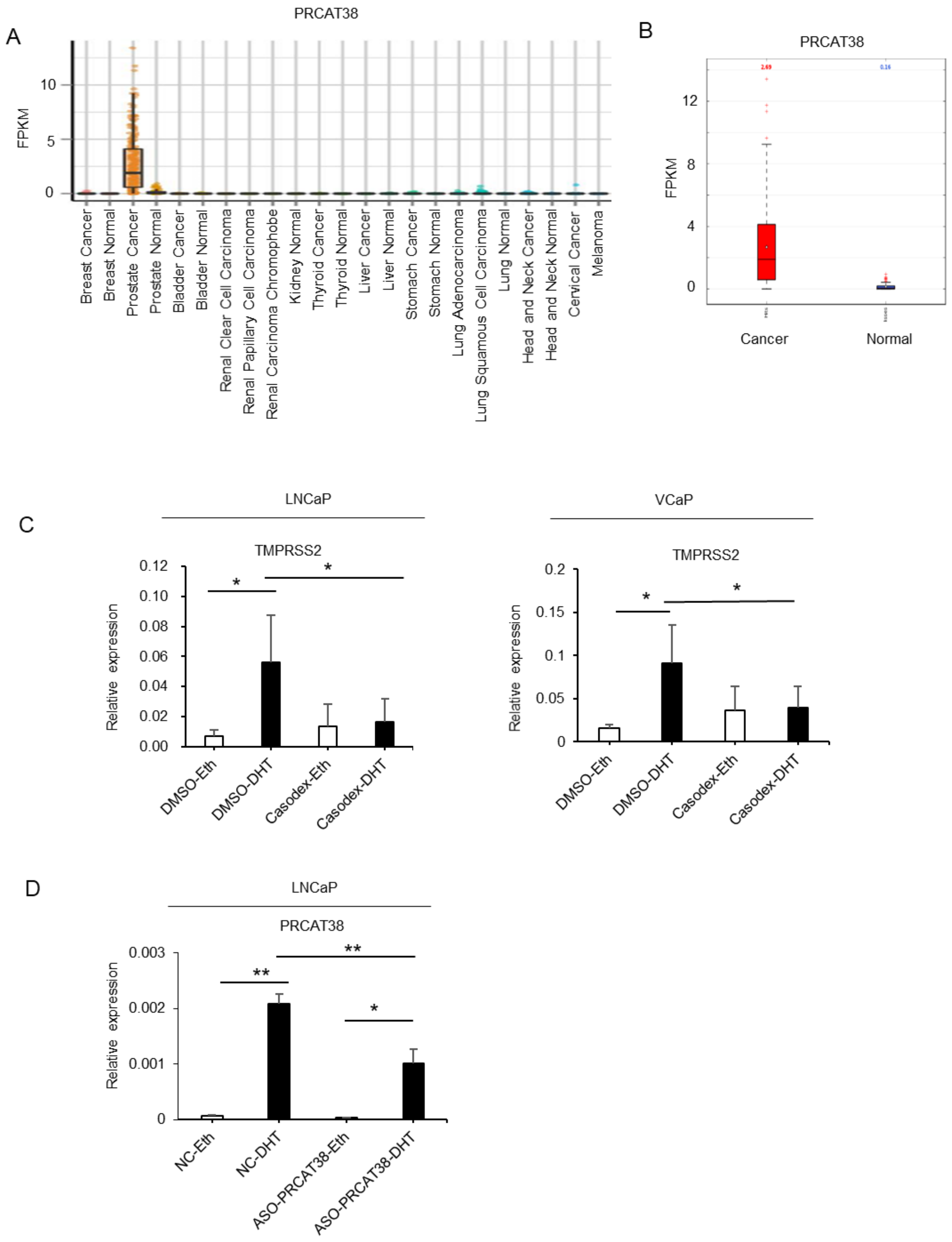

Supplementary Figure S1. *PRCAT38* is androgen responsive and specifically expressed in prostate cancer samples. A, expression level of *PRCAT38* across different tissue samples. B, *PRCAT38* is highly expressed in cancer as compared to normal prostate samples. The data are adapted from MiTranscriptome (www.mitranscriptome.org). C, androgen induction of *TMPRSS2* is inhibited by the androgen antagonist bicalutamide in LNCaP and VCaP cells. D, ASO-mediated knockdown of *PRCAT38* in LNCaP cells. Data are shown as the mean± SD (n=3). *: *P*<0.05, **: *P*<0.01, ns: not significant.

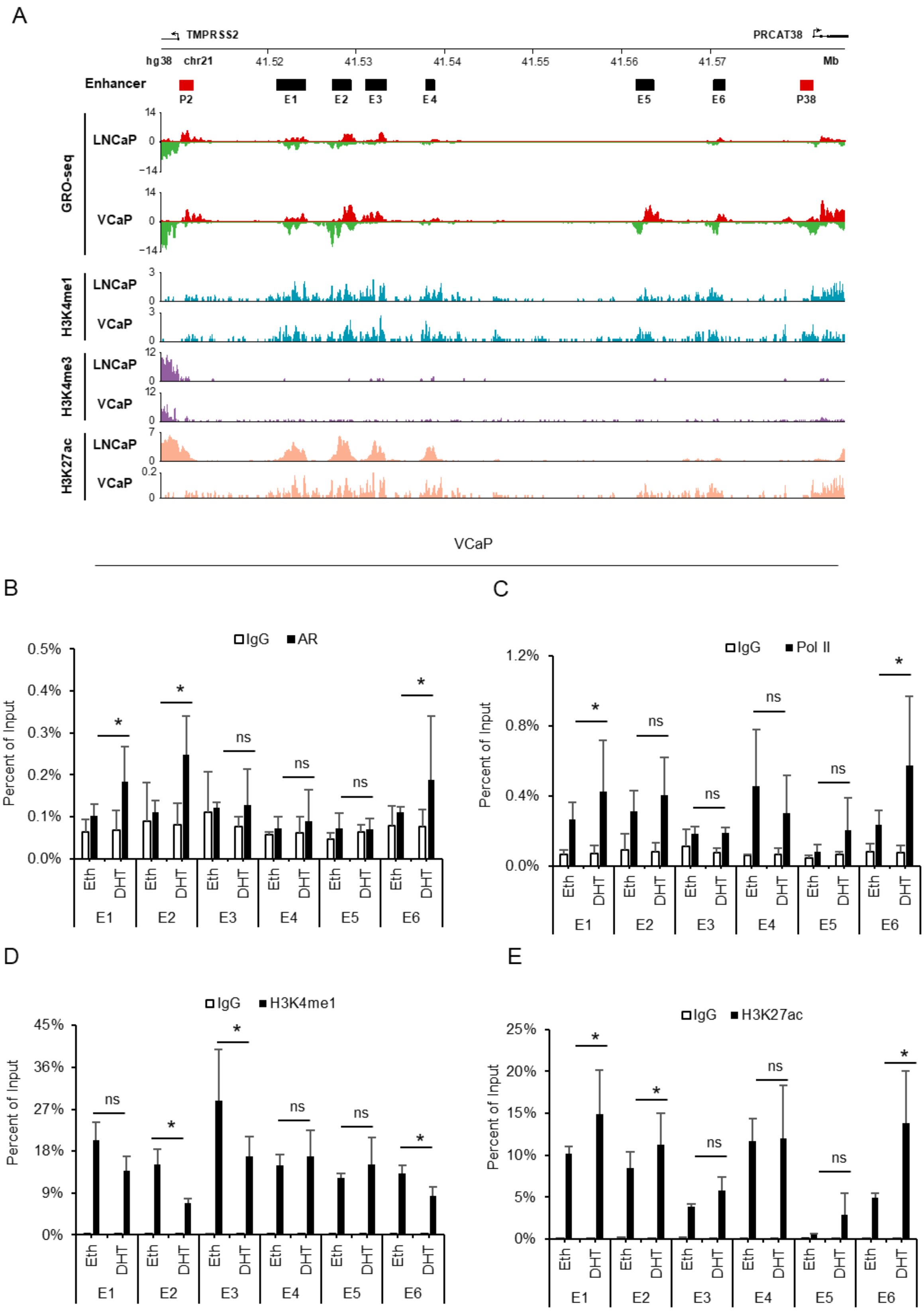

Supplementary Figure S2. AR binds to enhancers between *TMPRSS2* and *PRCAT38*, recruiting Pol II and H3K27ac to activate enhancers in VCaP cells. A, Identification of *PRCAT38* enhancers by integration of GRO-seq and ChIP-seq. B-E, ChIP-qPCR in VCaP cells before and after DHT treatment

showing AR, RNA polymerase II, H3K4me1 and H3K27ac enrichment over the enhancer regions. Data are shown as the mean± SD (n=3). *: $P<0.05$, **: $P<0.01$, ns: not significant.

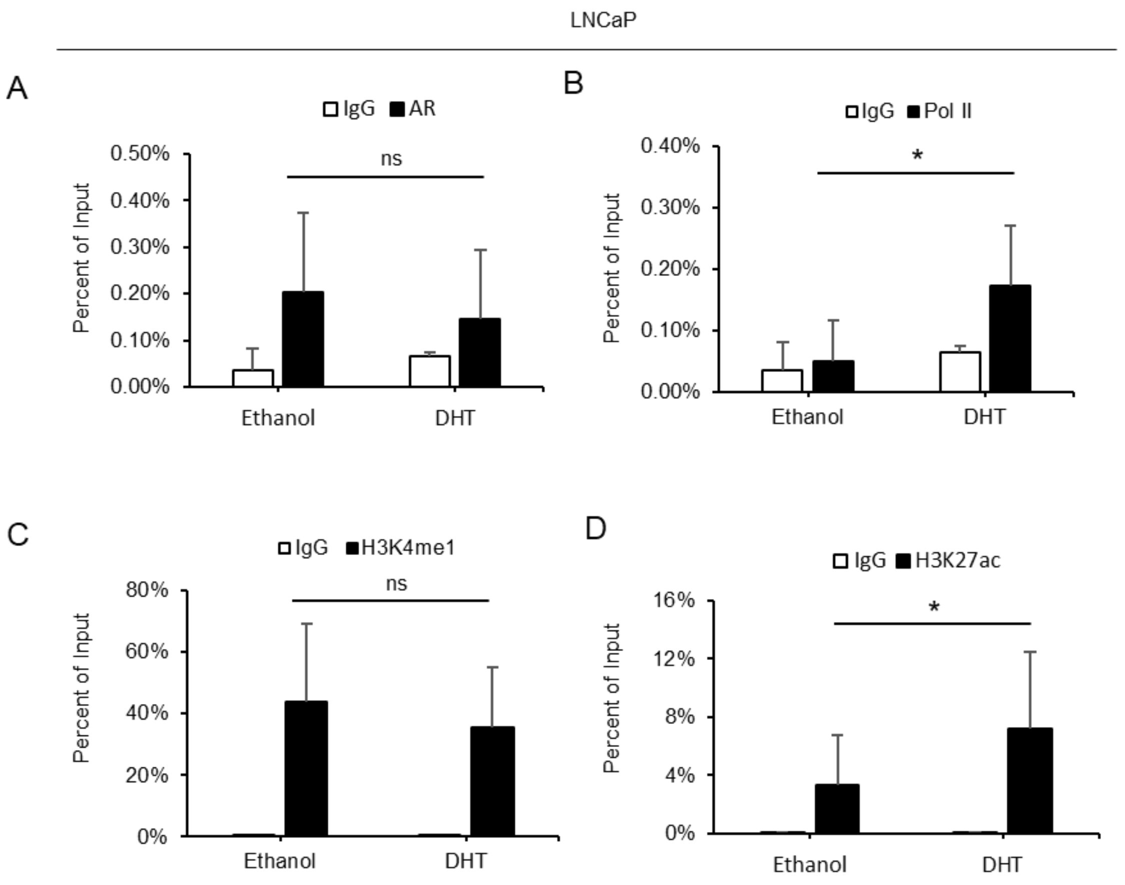

Supplementary Figure S3. AR, Pol II, H3K4me1 and H3K27ac enrichment at the *PRCAT38* promoter. A-D, ChIP-qPCR in LNCaP cells before and after DHT treatment showing AR, RNA polymerase II, H3K4me1 and H3K27ac enrichment over the *PRCAT38* promoter. Data are shown as the mean± SD (n=3). *: *P*<0.05, **: *P*<0.01, ns: not significant.

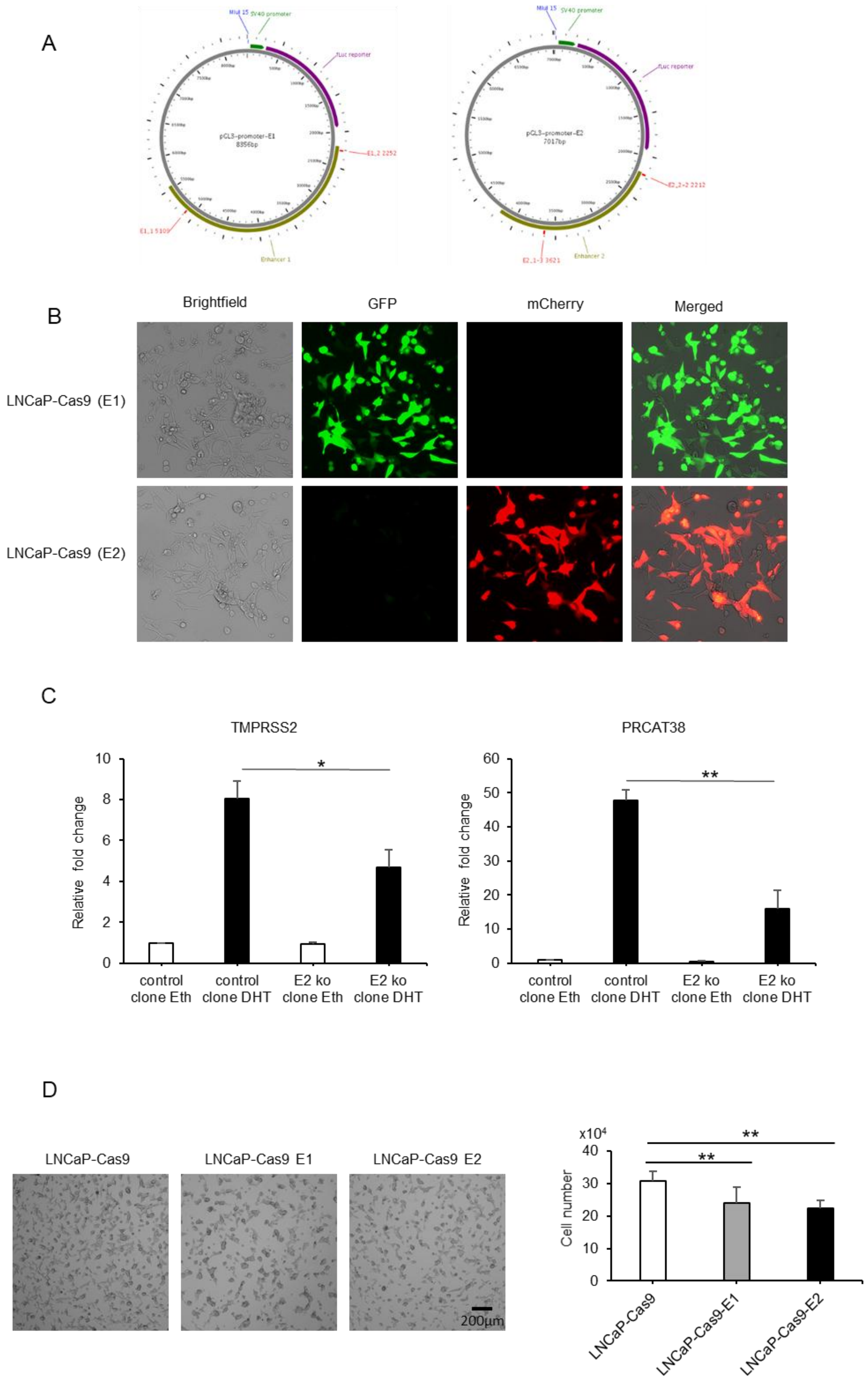

A
MluI 15
SV40 promoter
fLuc reporter
pGL3-promoter-E1
8356bp
E1_2 2252
E1_1 5109
Enhancer 1
MluI 15
SV40 promoter
fLuc reporter
pGL3-promoter-E2
7017bp
E2_2-2 2212
E2_1-3 3621
Enhancer 2
B
Brightfield
GFP
mCherry
Merged
LNCaP-Cas9 (E1)
LNCaP-Cas9 (E2)
C
TMPRSS2
Relative fold change
0
2
4
6
8
10
*
control clone Eth
control clone DHT
E2 ko clone Eth
E2 ko clone DHT
PRCAT38
Relative fold change
0
10
20
30
40
50
60
**
control clone Eth
control clone DHT
E2 ko clone Eth
E2 ko clone DHT
D
LNCaP-Cas9
LNCaP-Cas9 E1
LNCaP-Cas9 E2
200μm
x10⁴
Cell number
0
10
20
30
40
**
**
LNCaP-Cas9
LNCaP-Cas9-E1
LNCaP-Cas9-E2

Supplementary Figure S4. Knockout of enhancer E1 or E2 downregulates transcription of *TMPRSS2* and *PRCAT38*. A, Plasmids containing enhancer E1 or E2 as templates in *in vitro* assays (plasmids were drawn with a web tool “PlasMapper”). B, Transfection of dual-sgRNAs containing plasmids with GFP/mCherry reporter genes confirmed by fluorescence microscopy. C, Homozygous knockout clone for enhancer E2 shows downregulated *TMPRSS2* and *PRCAT38* transcription after DHT treatment as compared to the control clone. The control clone was derived from a single cell after the same knockout procedure but contains no deletion. D, Knockout of enhancer E1 or E2 impairs the proliferation of LNCaP cells. Data are shown as the mean± SD (n=3). *: P<0.05, **: P<0.01, ns: not significant.

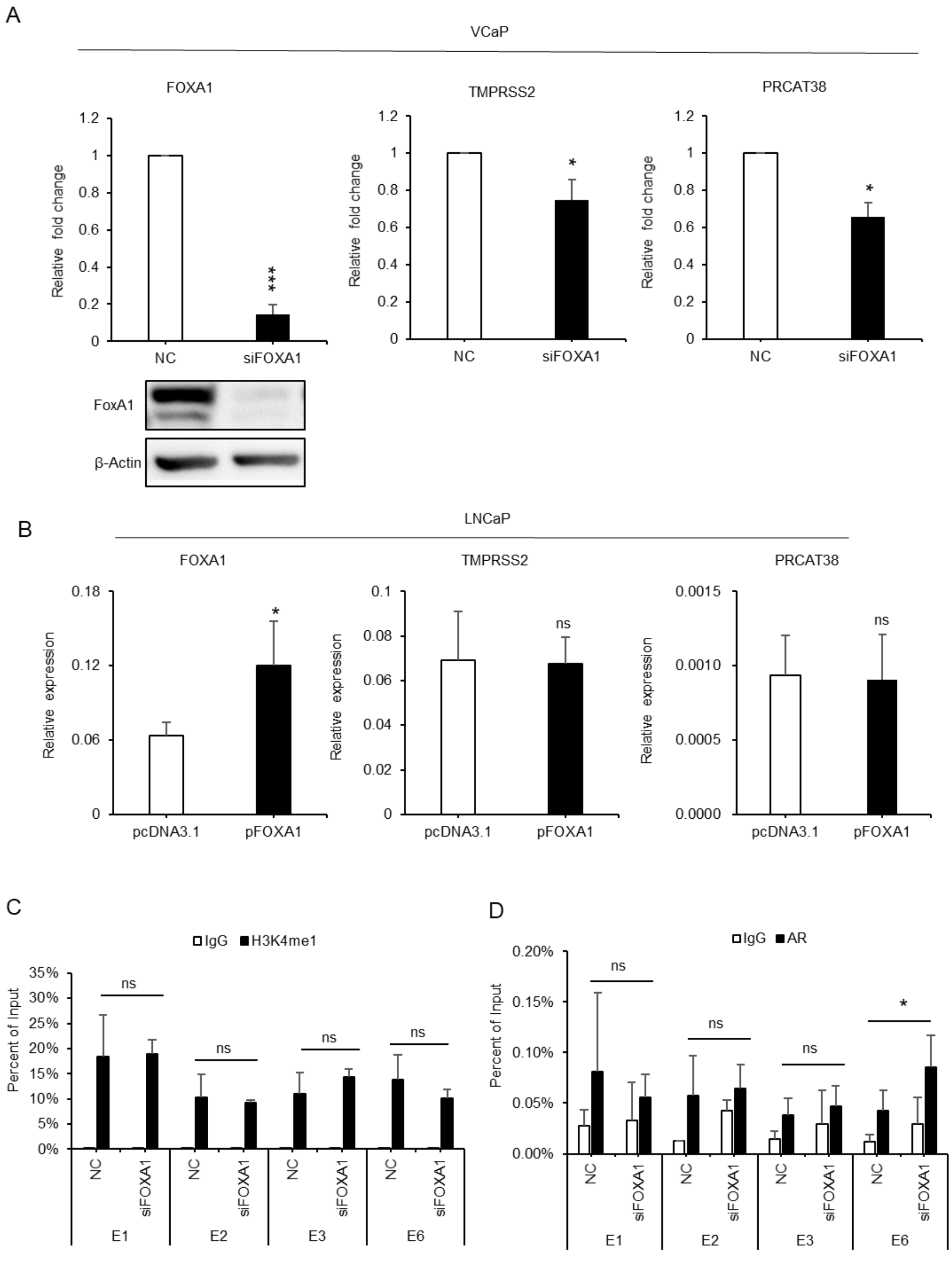

Supplementary Figure S5. Knockdown of FOXA1 inhibits *PRCAT38* transcription. A, qPCR and western blot showing down-regulation of *FOXA1*, *TMPRSS2* and *PRCAT38* after FOXA1 knockdown in VCaP cells. B, qPCR detection of *FOXA1* and *PRCAT38* transcription after FOXA1 overexpression. D-E, ChIP-qPCR in LNCaP cells before and after FOXA1 knockdown showing AR and H3K4me1 enrichment over the enhancer regions. Data are shown as the mean± SD (n=3). *: *P*<0.05, ns: not significant.

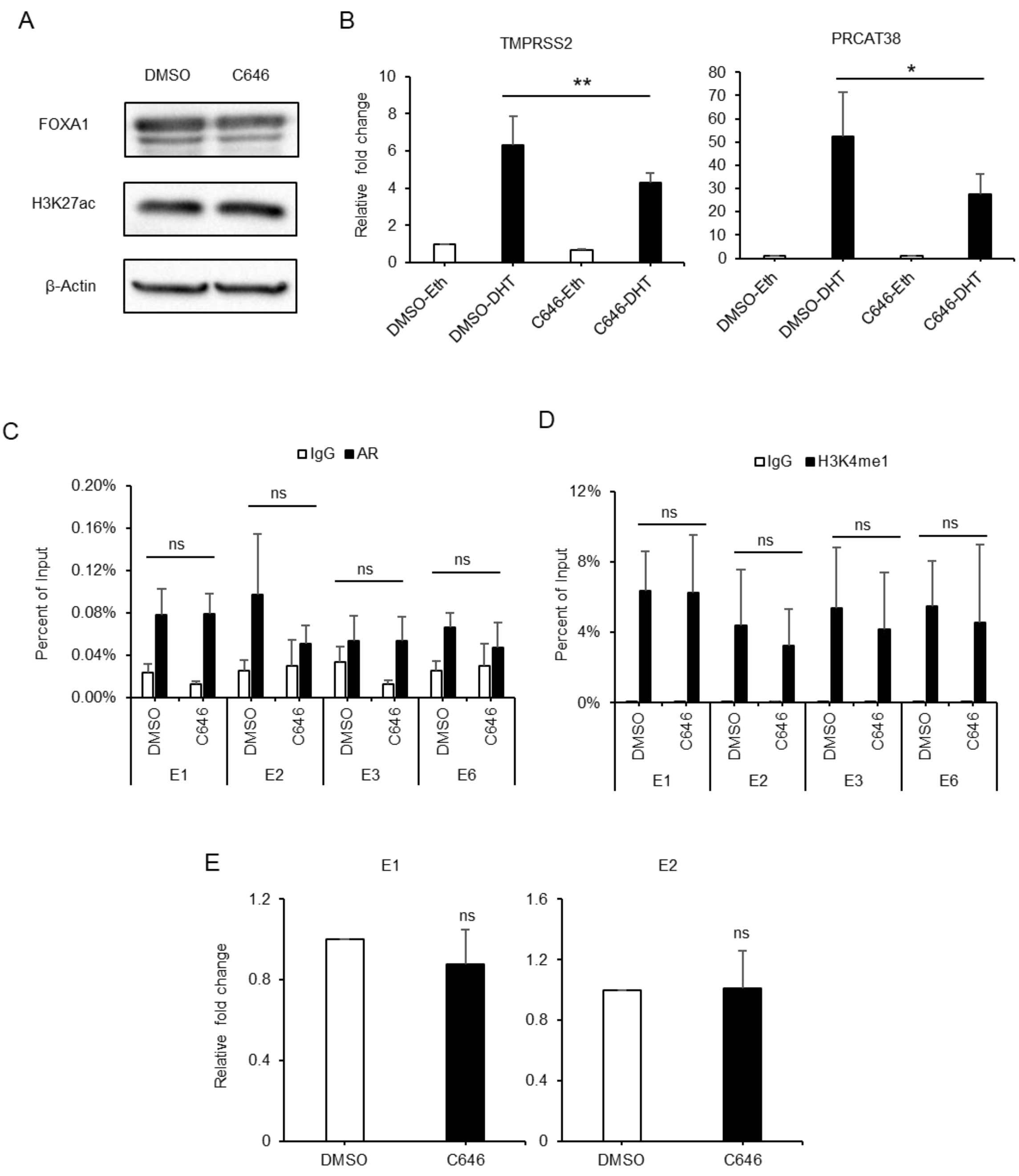

Supplementary Figure S6. p300 inhibition does not affect the total amount of H3K27ac or eRNA transcription from enhancer E1 or E2. A, Western blot showing the protein level of FOXA1 and H3K27ac before and after C646 treatment. B, transcription of *TMPRSS2* and *PRCAT38* before and after C646-DHT treatment. C-D, ChIP-qPCR in LNCaP cells before and after p300 inhibition showing AR and H3K4me1 enrichment over the enhancer regions. E, eRNA transcription levels from enhancer E1 and E2 before and after C646 treatment. Data are shown as the mean± SD (n=3).

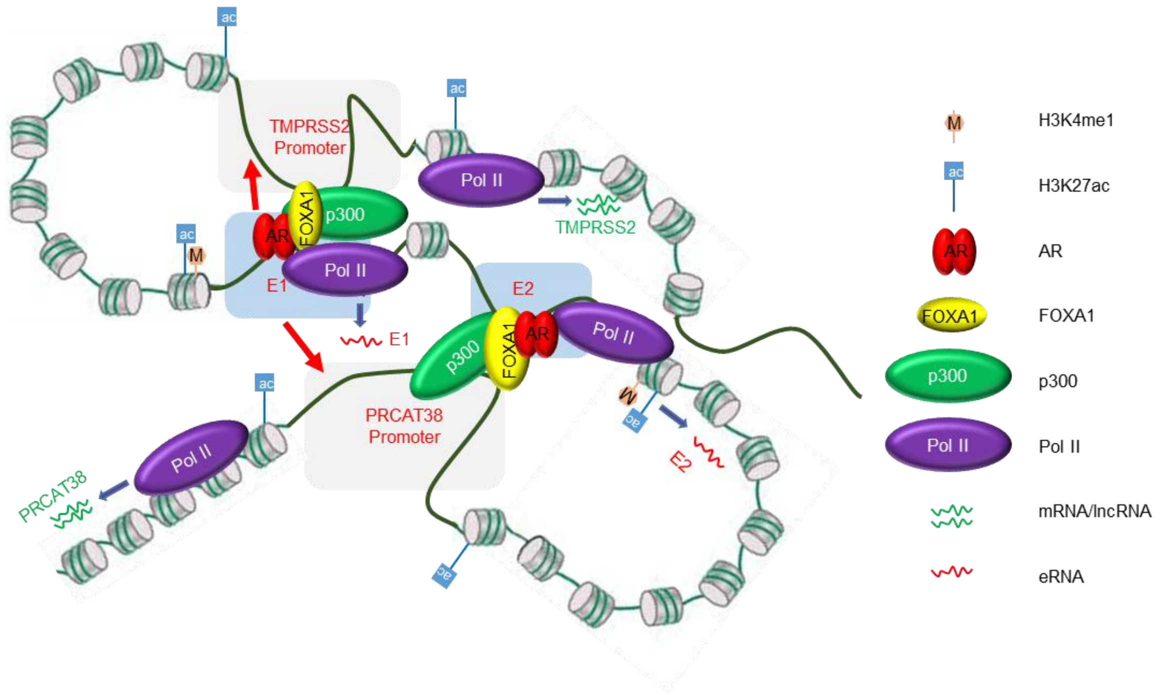

Supplementary Figure S7. Schematic for simultaneous regulation of *TMPRSS2* and *PRCAT38* by enhancer E1 and E2. E1 directly interacts with the *TMPRSS2* promoter while indirectly regulating *PRCAT38* with the aid of E2-*PRCAT38* promoter interaction.
